# Amplification of the basic reproduction number in cattle farm networks

**DOI:** 10.1371/journal.pone.0191257

**Published:** 2018-04-19

**Authors:** Beatriz Vidondo

**Affiliations:** Veterinary Public Health Institute, University of Bern, Bern, Switzerland; International Nutrition Inc, UNITED STATES

## Abstract

The popularly known 20–80 rule or Pareto rule states that 20% of efforts leads to 80% of results. This rule has been applied to the study of infection transmission in contact networks, and specifically, contact networks between cattle farms. Woolhouse and collaborators showed that targeting interventions for disease control and prevention to the 20% of the farms that contribute the most to the basic reproduction number (Ro), could reduce it by 80%. The rule results from the number of incoming and outgoing contacts per farm being highly heterogeneous. Besides, Woolhouse and collaborators showed that this high contact heterogeneity, together with a high positive correlation between the number of incoming and outgoing animal movements per farm leads to an amplification in the Ro. Two previous studies carried out with Scottish cattle transport data found either no correlation or only a weak correlation (rho up to 0.33) when using weighted data. Using data from the contacts between Swiss cattle farms in 2015, we found that the 20–80 rule applies with respect to Ro, although the proportion of highly active farms is smaller (11%). Besides, a positive strong correlation (rho = 0.64, weighted data) between the incoming and outgoing contacts of farms exists. This means that the amplification of Ro (due to the contact heterogeneities and the existing correlation) in cattle contact networks can be much higher than known until now. Our results highlight the importance of an effective active surveillance, more so than in other countries were these amplification mechanisms are absent.

## Introduction

The popularly known 20–80 rule or Pareto rule states that 20% of efforts lead to 80% of results ([1]). This constitutes in principle a convenient situation when tackling control of infectious diseases. In fact, it has been shown that this rule applies to Scottish cattle ([2],[3]) and sheep farms ([4]) respectively, and the percentages of the rule vary depending on the type of farm.

Empirically, the 20–80 rule for cattle farms results from the heterogeneities in the contacts between farms, namely the high variance found in the number of animals arriving and leaving per holding and unit of time, with many holdings having only a few contacts and only a few holdings having a very big number of contacts ([5]). A contact between holdings is defined every time animals are unidirectionally moved from one farm to another. The contact network refers then to the cattle movements between holdings within a certain time period. As shown by ([6]), Ro in heterogeneous contact networks is amplified by its high variance. Considering Ro as “the number of secondary infected farms produced by a typical farm early in an epidemic” ([7]) when all farms are still susceptible, and since the contacts are unidirectional, Woolhouse and collaborators ([2]–[4]) proposed that Ro would be “proportional to the mean of the product of the number of sources from which they receive cattle” (called indegree in the social network analysis jargon) and the “number of destinations to which they send cattle” (also called outdegree). See also ([8]) for a more in depth discussion. The first (indegree) informs about the susceptibility or the probability to acquire disease from other farms and the second (outdegree) informs about the infectiousness, or how likely it is to pass infections to other premises. When these two quantities are 1) highly heterogeneous and present high variance and 2) positively correlated, an amplification in the value of Ro occurs as it can be shown that the higher the correlation, the higher the expected value of the product ([2]–[4]). Interestingly, only weak correlations (maximum rho = 0.33) have been found for the Scottish cattle farms, and only when using weighted data ([3]).

Switzerland is a small alpine country with a relatively high density of cattle farms per km^2^. With ca. 1.5 Mio heads at the end of 2015 ([9]), cattle is the major livestock species in Switzerland. The average number of animals in premises declared as cattle and pig holders is 35 animals per holding with the 10th percentile being only 2 animals and the 90th percentile 110 animals ([9]). Markets widely range from small and middle sized arenas to big often exhibitions or breeding contests with international presence. Their frequency can be biweekly to every four years, depending on the type of event. In the months of May to October every year, the traditional practice of cattle summering to high altitude pastures takes place. Switzerland maintains a freedom of disease status for most of the highly contagious diseases that might affect neighbouring countries ([10]). A previous Swiss study ([11]) reported a highly positive skewed (i.e. non-Gaussian and highly overdispersed) yearly indegree and outdegrees and median yearly ingoing and outgoing contact chains 4 orders of magnitude larger than those in Sweden ([12]). These characteristics make Swiss cattle network unique with respect to other European countries and are the reasons why we chose it as a study area.

The statistical distributions of some European cattle contact networks such us in France ([13]) and Denmark ([14]) have been described as power-law or scale-free (i.e. characterized by a single exponent over many scales of degree, see also ([15])). The distribution of these broad scale social contact networks, however, can also be truncated for very large connectivity values due to aging or costs associated to large degrees ([16]). In the present study we present the connectivity distributions for the full cattle network (including markets and alps) and for farms only, and investigate 1) whether the 20–80 rule holds for Swiss cattle farms and 2) whether a correlation exists between the indegree and the outdegree as this would be indicative of amplifications in Ro for Switzerland.

## Materials and methods

The animal movement database (in German, Tier Verkehr Datenbank (TVD) or French, Banque de données sur le traffic des animaux (BDTA)) constitutes the nationwide registry for cattle and other animals in Switzerland. It records every holding and every individual bovine, its place and date of birth as well as every transport until its death ([17]). Animal transports from January, the 1st 2015 to December the 31st 2015 were extracted from the database using MS SQL Server. Only complete animal histories and unique records were included in the analysis. At the end of 2015 the percentage of records eligible for data analysis was above 99%. The number of animals transported every day from every premise of origin to every premise of destination was summed per day. For the present analysis, the end transports to slaughter and imports from and exports to abroad were excluded. With only 611 animals imported and 1336 animals exported out of a total of 3’563’424 total animal transports, the within-country contact network can be considered relatively closed. Seasonal transports to and from alpine pastures were included whereas daily commutes to common pastures at the valley near the farm of origin were excluded.

First the so-called edge lists (containing premise of origin, premise of destination, date of transport and number of animals transported) for all types of premises were prepared using SQL routines. Second, yearly indegree and outdegree (both weighted per the number of animals transported and unweighted) per holding were calculated using R package igraph (R version 3.1.2). Let *a*_*ij*_ be the direct contact rate from farm *j* to farm *i*, $\beta_{in}^{i}=\sum_{j}a_{ij}$ the yearly indegree for farm *i* and $\beta_{out}^{i}=\sum_{j}a_{ij}$ the yearly outdegree for farm *i*. In a closed network, ∑_*i*_ *β*_*in*_(*i*) = ∑_*i*_ *β*_*out*_(*i*)

Selecting farms only, and following ([3],[4]) we calculated Ro as the average of the product of the indegree and the outdegree, divided by the average indegree for all farms:

$$R_{0}=\frac{\frac{1}{N}\sum_{i=1}^{N}\beta_{in}\left(i\right)\beta_{out}\left(i\right)}{\frac{1}{N}\sum_{i=1}^{N}\beta_{in}\left(i\right)}=\frac{\frac{1}{N}\sum_{i=1}^{N}\beta_{in}\left(i\right)\beta_{out}\left(i\right)}{\frac{1}{N}\sum_{i=1}^{N}\beta_{out}\left(i\right)}=\frac{\bar{\beta_{in}\beta_{out}}}{\bar{\beta_{in}}}=\frac{\bar{\beta_{in}\beta_{out}}}{\bar{\beta_{out}}}$$

Where *β*_*in*_(*i*) and *β*_*out*_(*i*) refer to in- and out-contact rate, respectively, for farm *i*.

**Fig 1:**
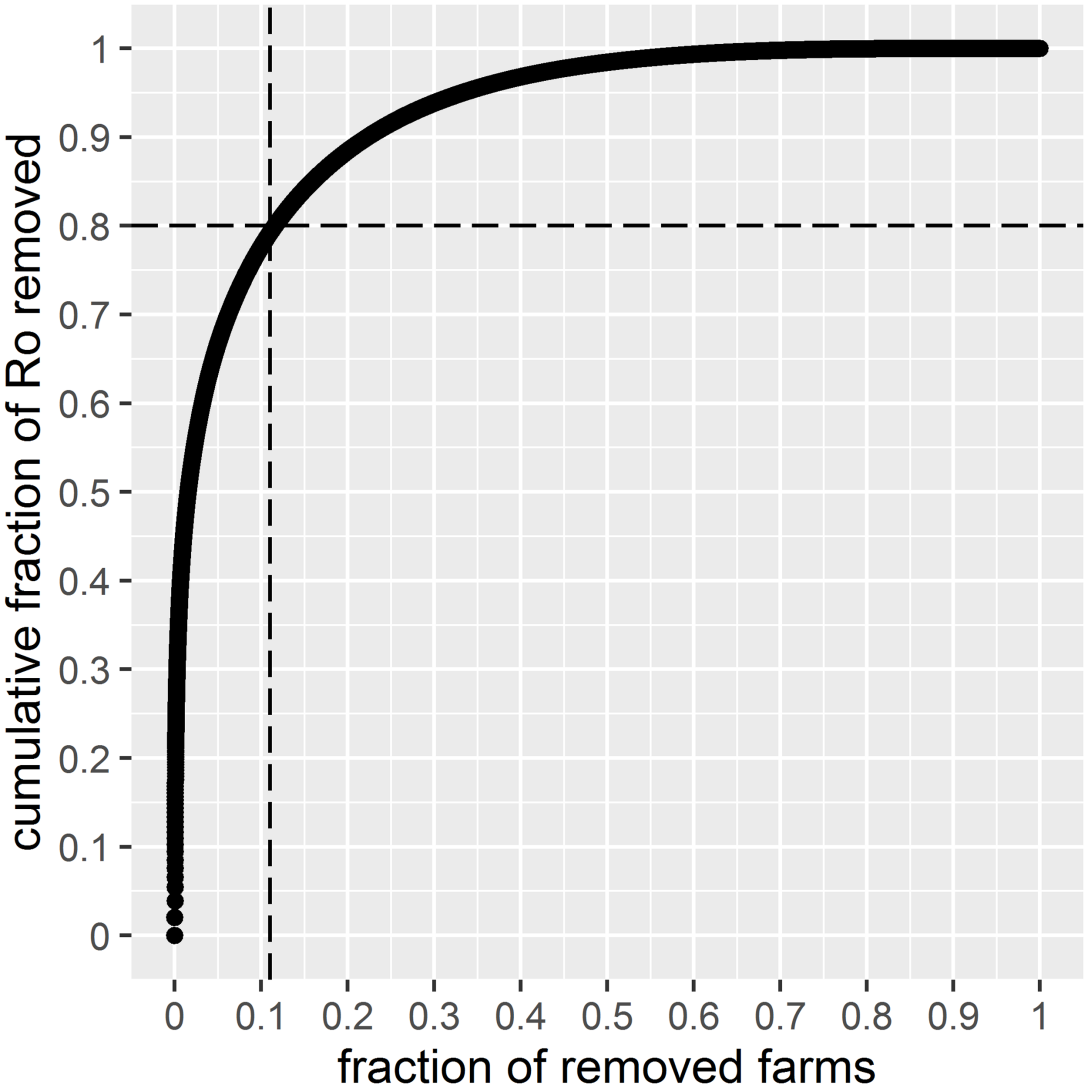
Decline in Ro when removing individual farms. Dashed vertical line corresponds to removal of 11% of holdings and the dashed horizontal line to 80% reduction of R0. Data for, Swiss farms only, 2015. Yearly indegree and outdegree have been weighted by the number of animals transported.

Fig 1 was created by ordering the farms from small to largest product indegree*outdegree and increasingly setting the value of the product of every farm to zero. The decline in Ro can be plotted against the percentage of farms removed. As explained in (2), this approach renders relative, not absolute, measures of the magnitude of Ro, and by aggregating over one year, the conclusions apply to a slow transmitting and evolving disease rather than to a fast and acute one. Spearman-rank correlation coefficients of the indegree and outdegree (both weighted and unweighted) were calculated for farms only, because farms are the holdings that keep animals permanently and are subject to surveillance and control measures. This is also the reason why some of the Figures contain farms only.

The empirical distributions of the indegree, outdegree (weighted and unweighted) for both the full network and farms only were inspected first visually by plotting survival distributions (1-P(x)) where P(x) is the cumulative distribution function. The empirical distribution of the standardized degree product *β*_*in*_(*i*)*β*_*out*_(*i*)/*mean*(*β*_*in*_) for farms only was also investigated. From the survival plots, it was apparent that different regimes were present along the gradient of degrees. Following ([16],[18]) we considered them as spliced distributions, that is, obeying different models for different ranges of degree. With this idea in mind, different models (Pareto (power-law), LogNormal and Exponential) were fitted to different ranges of degrees following the methods described by ([19],[20]) and using R package poweRlaw. The degree β obeys a power-law if it is drawn from a density distribution function of the kind $p(\beta )=\frac{\alpha -1}{\beta_{min}}({\frac{\beta }{\beta_{min}})}^{-\alpha }$ where α is a constant or the characteristic power-law exponent or shape parameter, and *β*_*min*_ is the minimum degree for which the power-law holds, or scale parameter. For all models, first *β*_*min*_ was estimated using a Kolmogorov-Smirnov approach ([20]) and then α or the corresponding parameters were estimated given that value of *β*_*min*_ using a maximum likelihood estimator ([19]). Bootstrapping was used to generate a p value that informs on the goodness-of-fit to the corresponding model. A significant p-value suggests significant deviations from the model considered. The estimation of p-values by bootstrapping is resource intensive and had to be parallelized and carried out on a computer cluster.

## Results

We found that the Pareto rule holds for Swiss farms with a very similar picture as those published in ([2]) (their Fig 1) and ([3],[4]) (their Fig 3). The removal of 11% of the holdings leads to a Ro reduction of 80% (Fig 1) for both the weighted and unweighted data. It is remarkable that 50% of the Ro can be removed by excluding a small percentage of farms.

**Fig 2:**
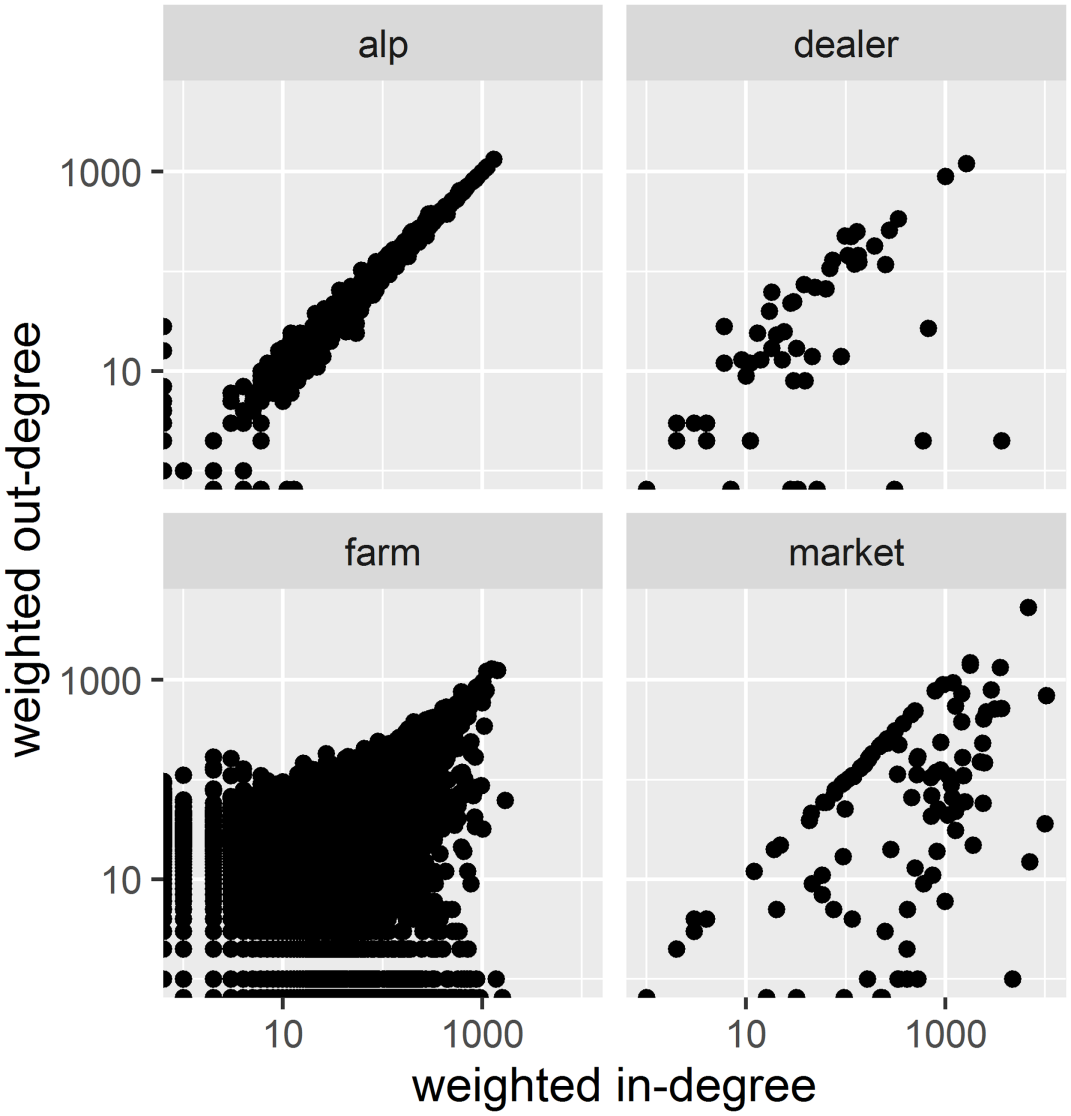
Scatter-plots of indegree versus outdegree (weighted data). Holding categories from left to right in alphabetical order: alp (holding at high altitude to access summer pasture), dealer, farm, market (including trade markets, auctions and exhibitions). Vertical axis: outgedree. Horizontal axis: indegree. Yearly weighted degrees are presented on a double logarithmic plot.

Besides, we found a positive correlation between yearly indegree and outdegree for Swiss farms and this correlation is much higher for weighted data (Spearman-rank correlation coefficient, rho = 0.64, p-value <0.0000) than unweighted data (rho = 0.25, p-value <0.0000). To present a more complete picture, the scatterplots of indegree versus outdegree not only for farms but also alps, markets and dealers are also shown in Fig 2 (weighted data only).

For all categories, the association tends to follow the 1:1 line in double logarithmic plot, that is a relation of the form indegree = outdegree. A perfect 1:1 line means that for every ingoing animal transport, an outgoing transport occurs at the yearly scale. For alps the scatter around the 1:1 line is minimal as most of the animals enter the alps in Spring and leave to the farm of origin in Autumn.

For farms the association between indegree and outdegree occurs more clearly for values above 100, that is, for the most active holdings. The markets show additionally quite a significant scatter below the 1:1 line. Markets with a high indegree and a low outdegree are most probably slaughtermarkets, markets where the animals are priced for slaughter ([11]). The low outdegree is a consequence of having excluded the transports to slaughter (see Methods).

**Fig 3:**
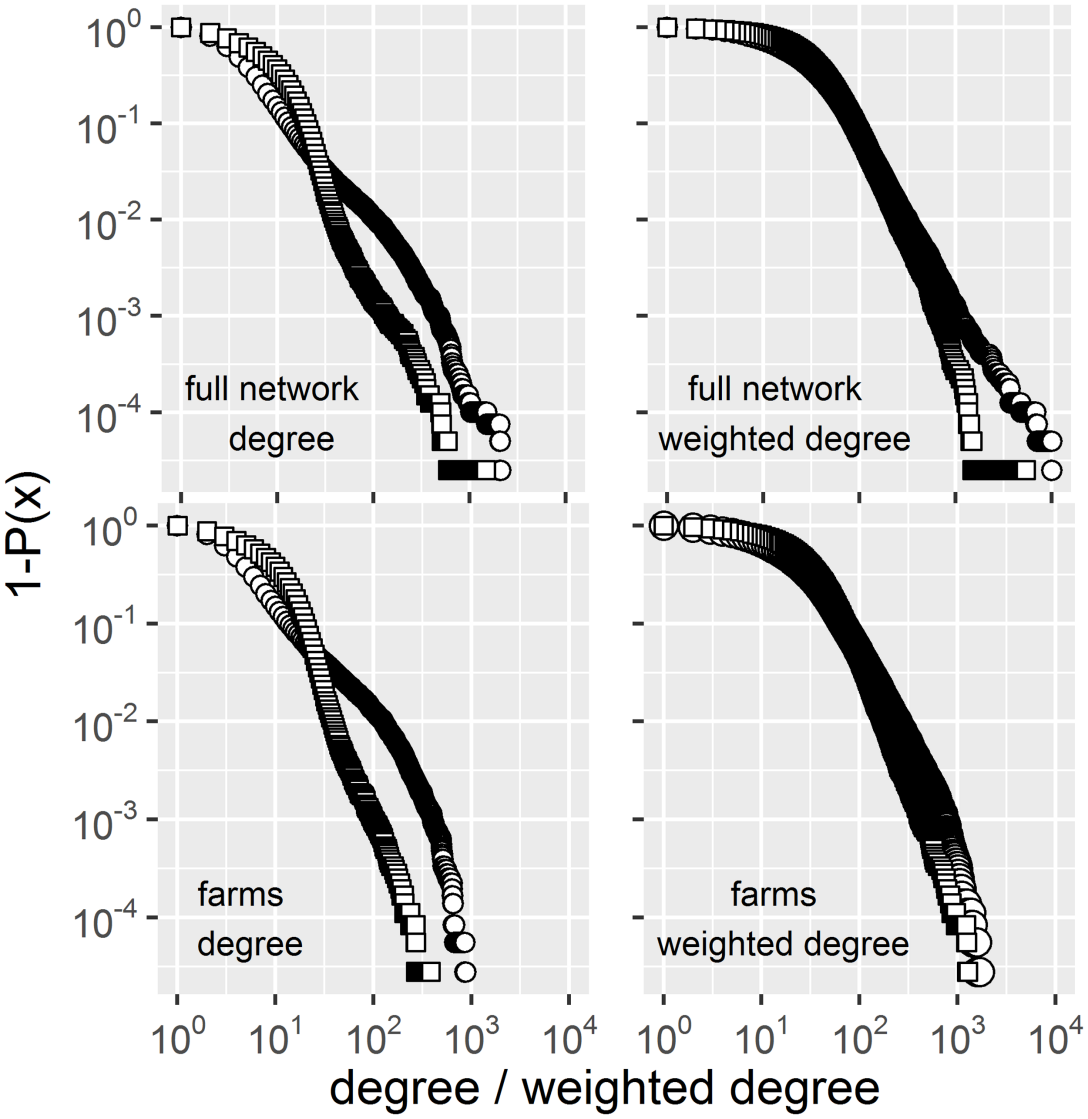
Log-Log plots of the survival distributions (1-P(x)). Both unweighted and weighted degree for the full network and farms only are presented. Indegree (circles) and outdegree (rectangles). All plots show highly overdispersed distributions with different regimes along the gradient of degree.

**Fig 4:**
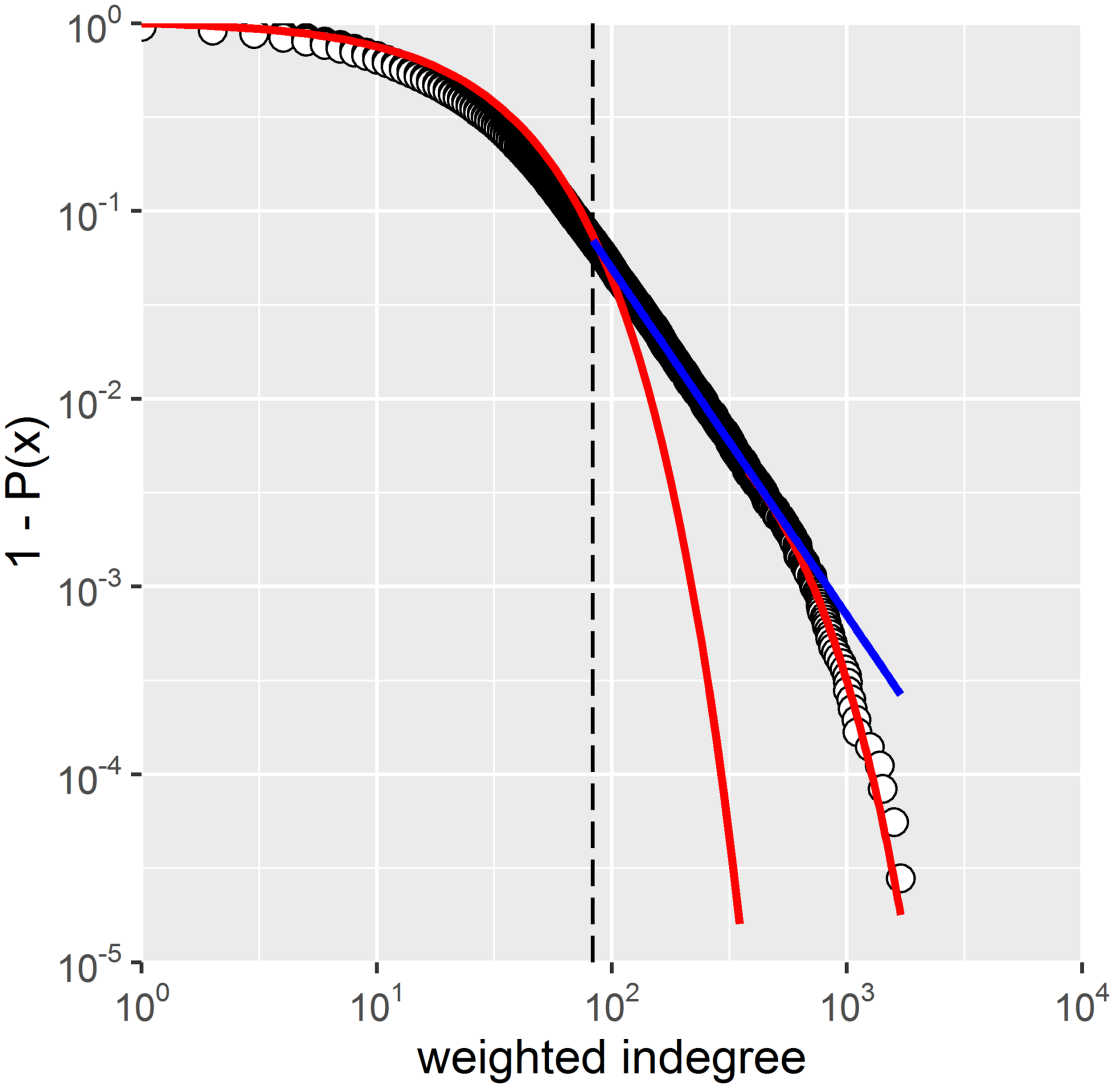
Log-Log plot of the survival distribution of weighted indegree for farms only. A spliced distribution trend is present. Blue line: power law model with *x*_*min*_ = 83 (vertical dashed line) and α = 2.85 (n = 2’489 farms, p-value = 0). Red lines: 1) Exponential model with *β*_*min*_ = 1 fitted for all active farms (n = 35’733, p-value = 0.85). This red line is presented for the whole range as a guide to the eye of what an Exponential decay would be; 2) Exponential model with *β*_*min*_ = 380, n = 146 farms, p-value = 0.75.

**Fig 5:**
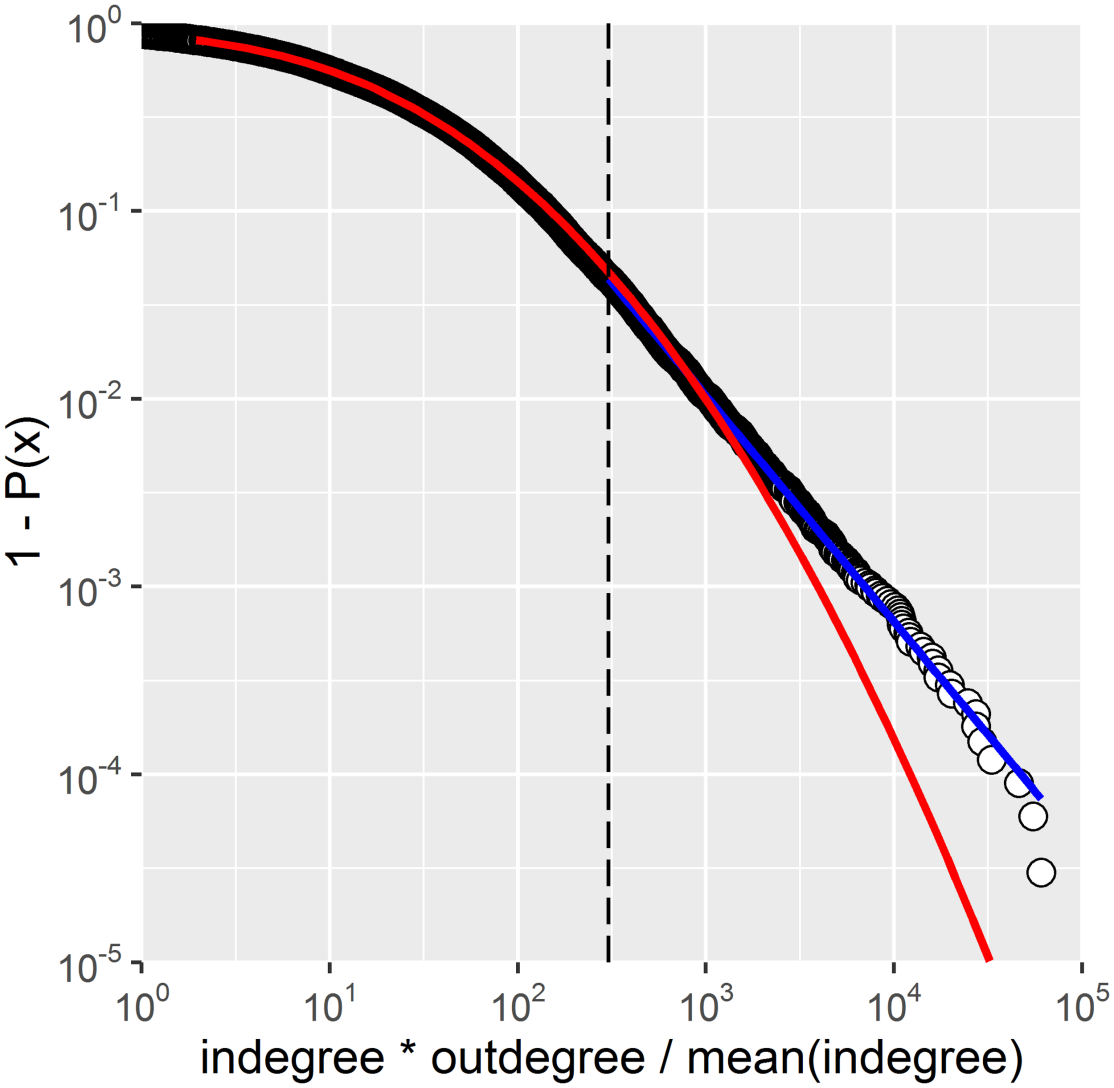
Log-Log plot of the survival distribution of the standardized degree product. Farms only (weighted data). A spliced distribution trend is present. Blue line: power law model with *x*_*min*_ = 303 (vertical dashed line) and α = 2.2 (n = 1’461 farms, p-value = 0.09). Red line: LogNormal model with *x*_*min*_ = 1.95, n = 27’125 (out of a total of 33’350 farms, i.e. farms with both indegree and outdegree > 0, p-value = 0). The LogNormal model is presented for the whole range as a guide to the eye of what the LogNormal would be.

The empirical distributions of the indegree and the outdegree (unweighted and weighted) for farms only and the full network (Figs 3 and 4) as well as that of their product (Fig 5) extend over three, four and up to five orders of magnitude, respectively. These broad scale distributions present different regimes along the gradient of degrees. Model fits for weighted indegree and outdegree are presented in Fig 4 (farms only) and S1 Fig, respectively. Model fits for unweighted data can also be found in the supplementary material.

As an example, weighted indegree of farms showed three regimes that partially overlapped with each other: 1) an exponential decline for lower connectivity values, 2) followed by a regime close to a power law (linear trend in this log-log plot) and 3) exponential decline for very large degree values (Fig 4). The middle range 83< *β* < 380 fit to a power law (blue-line) (*β*_*min*_ = 83, α = 2.85, n = 2’489 farms) did not converge (p-value = 0 for a bootstrap sample of 100 simulations). The Exponential fits (red lines) did converge for *β*_*min*_ = 1, p-value = 0.85, n = 35’733 farms and *β*_*min*_ = 380, n = 146 farms, p-value = 0.75). The first Exponential model, however, visually departs from the data around *β* > 83. The proportion of farms (1 ≤ *β* < 83 *and β* > 380) that follows Exponential distributions represents with 93% the majority of the total number of active farms. The weighted outdegree (S1 Fig) presented only two regimes (Exponential 3 ≤ *β* < 100 and power-law *β* > 100).

The standardized degree product was consistent with a LogNormal distribution with *x*_*min*_ = 1.95 (Fig 5, red line) but only up to x~303, value above which a power-law could be fit (Fig 5, blue line, *x*_*min*_ = 303, α = 2.2, p-value = 0.09). Both models visually overlap for 303 < x < 1’000. The number of observations to which the power-law could be fit was only 4.4% (1’461 farms out of a total 33’350 active farms for which the standardized degree product was larger than zero).

## Discussion

Our results show that the Pareto rule, previously described for Scottish farms, also holds for Swiss cattle farms and this is indicative of high overdispersion in their contact network. This, together with the found strong positive correlation between indegree and outdegree means that Ro would be potentially amplified in the case of a disease outbreak.

Our analysis was carried out in a similar way as the one presented by ([2]–[4]) and allows us to directly compare with their results. The strong correlation for Swiss cattle holdings categorized as ‘farms’ makes this country probably unique in the European landscape, although this finding would have to be confirmed by future studies. Spearman-rank correlation coefficient is adequate for this type of monotonic non-linear association and is not affected by the presence of outliers. The correlation was robust even after having corrected some holding’s categories (clinics and markets erroneously classified as farms). Holding’s classifications are thus not to be taken blindly and need to be double checked with any other data available.

Fitting statistical distributions to highly overdispersed data is not a trivial exercise and it should be done according to specific goals and considering additional information when available ([18]). The model fits presented here are thus intended as a guide only. Low and significant p-values as those obtained for the power-law fits in our study have been described before ([19]) and suggest that significant deviations from scale-free behaviour exist for Swiss cattle farms. The existence of different regimes along the gradient of connectivity (indegree and outdegree) of farms suggests the existence of varying underlying control phenomena governing the contact structure. Using a mathematical model, Amaral and collaborators ([16]) have proposed the aging of the nodes and the costs associated to very large connectivity as possible mechanisms to truncate scale-free contacts and give rise to Exponential decays in the contact structure. These mechanisms could be at work in the contact structure of Swiss farms.

The empirical distribution of the standardized degree product is not only highly overdispersed but also shows different regimes from minimum to maximum. Our results suggest that an arithmetic mean (the expected value of a Gaussian process) is not the best way to estimate its expected value. A better estimate of its first and second moments (expected value and variance) could be the scope of a future study.

Nevertheless, the product of indegree and outdegree is quite simple to calculate and can be used as a criterion for surveillance and control measures. Our results coincide with those in ([3],[4]): Ro amplification (also called multiplicative effect therein) increases when considering indegree and outdegree weighted by the number of animals transported. In the case of the Swiss cattle farms, this is even more relevant due to the presence of a strong correlation between the two. The logical consequence is that an effective reduction in the amplification of Ro can be achieved by two ways, reducing the variance and reducing the correlation. A reduction of the variance can be achieved by limiting the maximum number of transports per year and also, by limiting the maximum number of animals transported per batch. Tackling the correlation requires specific knowledge on the contact network in order to understand why this correlation exists. One possible reason is the existence of specialized farms (usually located in the central lowlands) where young animals are reared and return to the farm of origin or are sold to different farms. Another reason is the participation in the alpine summering although it has been estimated that only 40% of the Swiss farms do so ([11]). Specific interventions and their effect in reducing Ro would need to be quantified using models of disease spread.

In conclusion, Ro in Swiss cattle farms is amplified by 1) heterogeneities in the contact structure and 2) the existing positive strong correlation between indegree and outdegree. Our results highlight the importance of an effective active surveillance, more so than in other countries were these amplification mechanisms are absent. For emergent diseases not included in the annual serological surveys such as Lumpy Skin Disease with fast spread in South-East Europe ([21]) our results support the implementation of zootechnical measures (quarantine and testing) to keep the risk of introduction low, as well as the elaboration of preparedness plans for the early application of intervention measures.

## Supporting information

S1 Fig Log-Log plot of the survival distribution of weighted outdegree for farms only. A spliced distribution trend is present. Blue line: power law model with *x*_*min*_ = 99 (vertical dashed line) and α = 3.65 (n = 1’611 farms, p-value = 0). Red line: Exponential model with *β*_*min*_ = 3 (n = 33’589, p-value = 0). This red line is presented for the whole range as a guide to the eye of what an Exponential decay would be.
(TIFF)

S2 Fig Log-Log plot of the survival distribution of unweighted indegree for farms only. A spliced distribution trend is present. Blue line: power-law model with *x*_*min*_ = 3, *x*_*max*_ = 100 and α = 2.37 (n = 21’973, p-value = 0). Red line: Exponential model with *β*_*min*_ = 273 (vertical dashed line) (n = 115, p-value = 0.42).
(TIFF)

S3 Fig Log-Log plot of the survival distribution of unweighted outdegree for farms only. A spliced distribution trend is present. Blue line: power law model with *x*_*min*_ = 51 (vertical dashed line) and α = 3.28 (n = 151 farms, p-value = 0.53). Red line: Exponential model with *β*_*min*_ = 1 fitted for all active farms (n = 35’733, p-value = 0.97). This red line is presented for the whole range as a guide to the eye of what an Exponential decay would be.
(TIFF)
