# Supplementary figures and images for: Amplification of the basic reproduction number in cattle farm networks
Source: PLoS One. 2018 Apr 19;13(4):e0191257. doi: 10.1371/journal.pone.0191257 (PMC5909513; doi:10.1371/journal.pone.0191257)

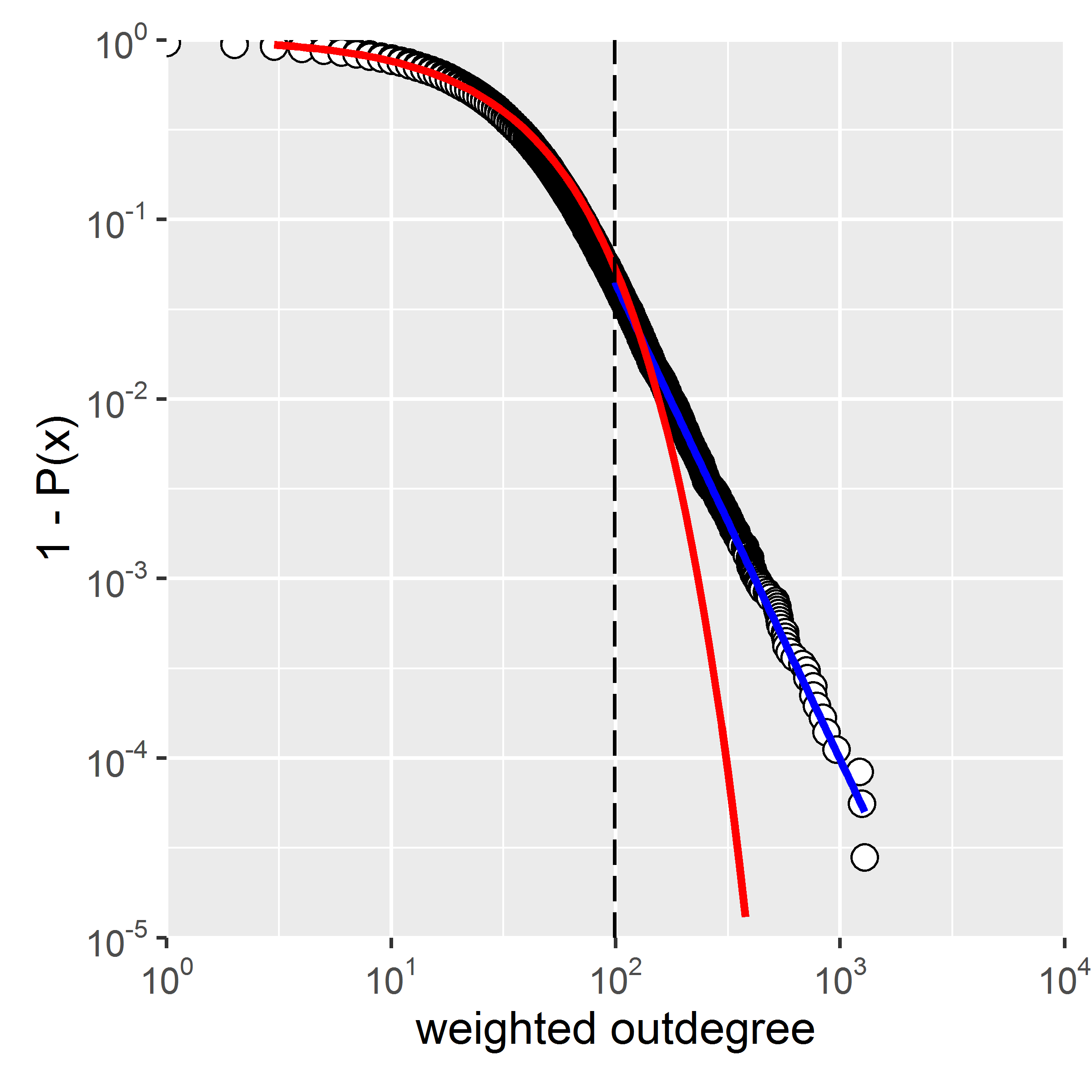

Supplement: S1 Fig — A spliced distribution trend is present. Blue line: power law model with xmin = 99 (vertical dashed line) and α = 3.65 (n = 1’611 farms, p-value = 0). Red line: Exponential model with βmin = 3 (n = 33’589, p-value = 0). This red line is presented for the whole range as a guide to the eye of what an Exponential decay would be. (TIFF) [file pone.0191257.s001.tiff]

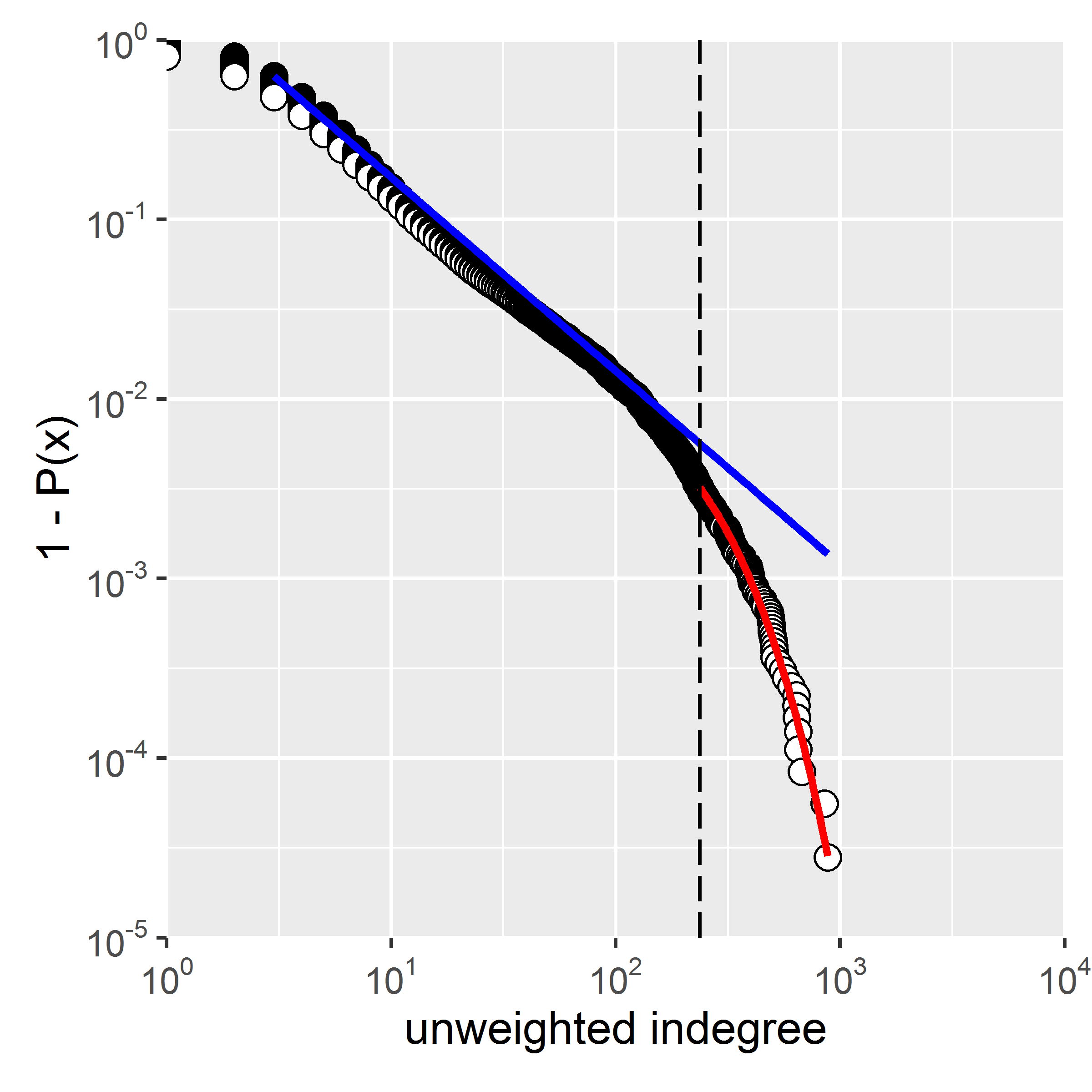

Supplement: S2 Fig — A spliced distribution trend is present. Blue line: power-law model with xmin = 3, xmax = 100 and α = 2.37 (n = 21’973, p-value = 0). Red line: Exponential model with βmin = 273 (vertical dashed line) (n = 115, p-value = 0.42). (TIFF) [file pone.0191257.s002.tiff]

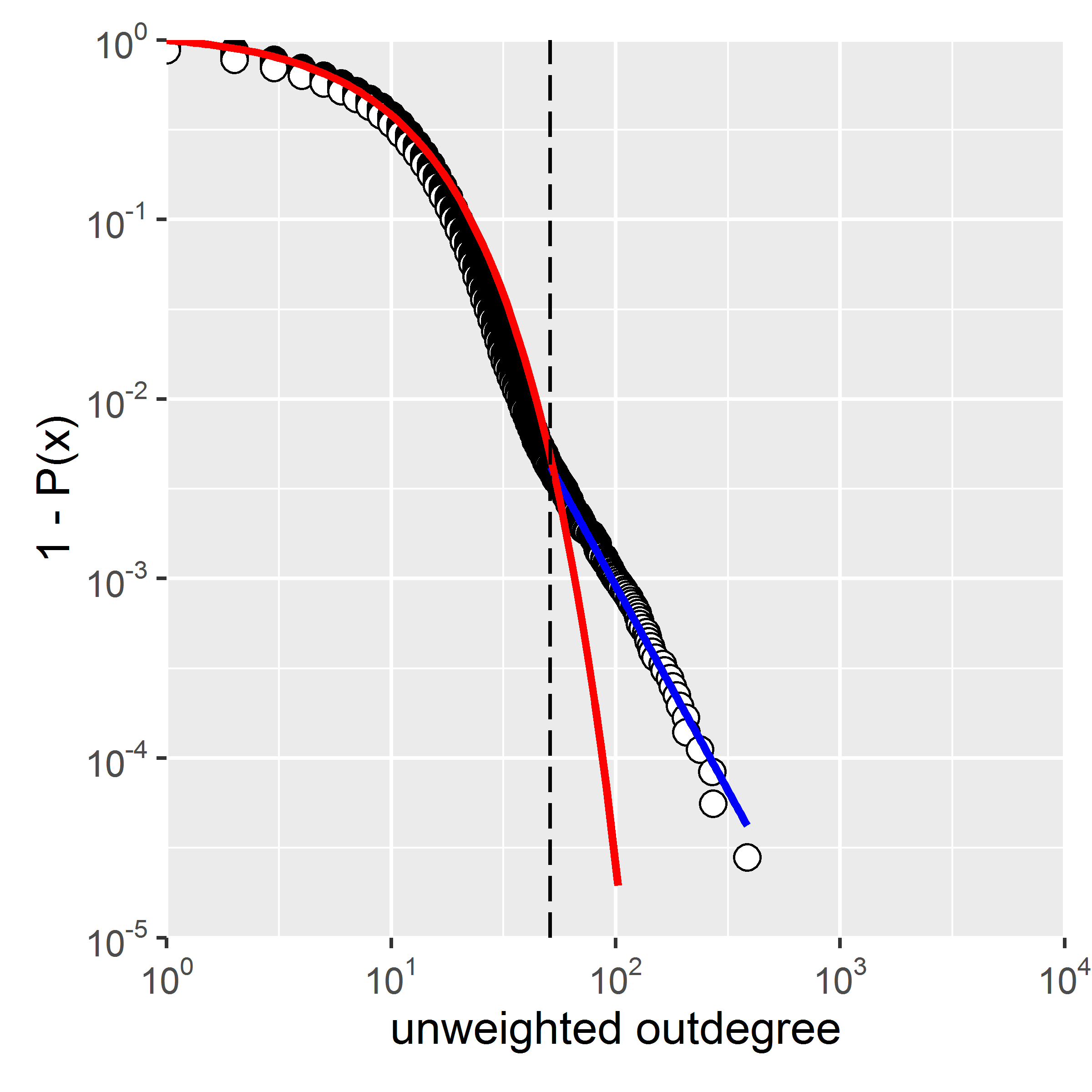

Supplement: S3 Fig — A spliced distribution trend is present. Blue line: power law model with xmin = 51 (vertical dashed line) and α = 3.28 (n = 151 farms, p-value = 0.53). Red line: Exponential model with βmin = 1 fitted for all active farms (n = 35’733, p-value = 0.97). This red line is presented for the whole range as a guide to the eye of what an Exponential decay would be. (TIFF) [file pone.0191257.s003.tiff]
